# Aliasing Artifacts in Multielemental LA-ICP-TOFMS Mapping: An Old Problem Revisited

**DOI:** 10.1021/acs.analchem.6c01023

**Published:** 2026-06-03

**Authors:** Filip Cernatič, Kristina Mervič, Lukas Schlatt, Martin Šala

**Affiliations:** † Department of Analytical Chemistry, National Institute of Chemistry, Hajdrihova ulica 19, SI-1001 Ljubljana, Slovenia; ‡ Department of Catalysis and Chemical Reaction Engineering, National Institute of Chemistry, Hajdrihova ulica 19, SI-1001 Ljubljana, Slovenia; § 390094 Nu Instruments, LL13 9XS Wrexham, U.K.

## Abstract

Recent works on quadrupole LA-ICP-MS have shown that unsynchronized laser pulses and data acquisition routines can produce pronounced signal aliasing. This phenomenon appears in the form of long-range and low- to medium-frequency alterations in time-averaged signals that do not reflect the actual underlying analyte concentration distribution, leading to wrong quantitation and considerably deteriorated image quality. In quadrupole instruments, total cycle times, settling and dwell times per nuclide could be finely tuned to match the pulse time of the laser (and/or SPR), thereby eliminating these artifacts. By contrast, in LA-ICP-TOFMS, exact synchronization between the two instrumental components is impossible even in principle. The reason lies in the fact that the acquisition time in the mass analyzer is a constrained value, and can only be set as an integer multiple of base acquisition time of the instrument. As a result, some level of aliasing is inevitable even if the acquisition time is set close to the SPR. In this work, we discuss the choice of optimal acquisition time in TOF instruments. Theoretical simulations and experimental mappings of standard reference materials strongly suggest that a compromise has to be made to minimize the spread of the signal without producing too much redundant data.

## Graphical abstract

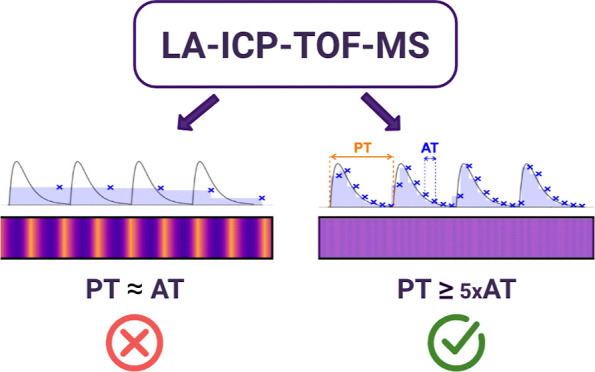

## Introduction

Over the last four decades from its introduction,^1^ laser ablation inductively coupled plasma mass spectrometry (LA-ICP-MS) has become a widely successful analytical technique for determining micro and trace elemental and isotopic constituents in samples.^2^ Owing to its microdestructive nature of sampling and negligible sample preparation workload, LA-ICP-MS is nowadays routinely utilized for spatially resolved elemental mapping of solid samples, having been adopted in a large variety of applications such as cultural heritage,^3^ forensics,^4^ geosciences^5^ and life sciences.^6^ The introduction of the time-of-flight (TOF) mass spectrometer,^7,8^ together with recent developments of low dispersion, rapid aerosol transport systems with short (<10 ms) single pulse response (SPR) time and fast data acquisition,^9–13^ have accelerated the development of multielemental mapping. By extending the capabilities of measurement to millions of pixels per hour, these technological advancements have consolidated LA-ICP-TOFMS as a state-of-the-art elemental mapping approach, capable of quasi-simultaneous determination of multiple high-resolution elemental maps covering large sample surfaces. In line with these hardware developments, considerable effort has been invested into the theoretical understanding and optimization of various parameters influencing image quality.^14–17^ The latter requires a delicate trade-off of competing constraints inherent to the measurement process, characterized by variables like the laser beam size, repetition rate (RR) and acquisition time (AT) of data from the MS detector and instrumental constants like Flicker noise and the shape of the SPR profile.^16^ In this work, we focus on the single-pulse analysis mode, which is the fastest mapping mode, where a single laser shot is applied per individual pixel and the signal detected from the SPR of each individual pulse is integrated per pixel window into the final spatially resolved elemental information. Numerous artifacts can negatively affect spatially resolved analysis, leading to inaccurate and unreliable information presented in elemental maps. For a fixed RR and AT, increasing the beam size leads to higher sensitivity and shorter mapping times, but inevitably leads to the loss of local details due to lower resolution. On the other hand, smaller beam sizes allow for finely resolved image details, up to a point where the Poisson noise deteriorates image quality due to poor counting statistics. A more subtle deleterious effect on image quality, spatial aliasing, which has received some attention in recent years, arises when the laser pulse (dependent on RR) and data acquisition intervals (characterized by AT) are unsynchronized.^16^ For a laser pulse time PT = 1/RR, this can happen in different settings, whenever one of the time intervals, PT or AT, is not an integer multiple of the other. The mismatch between these two time scales, when it occurs, leads to a long-range drift in the data acquisition, which manifests itself in low- to medium-frequency oscillations in the image. Several solutions have been proposed to alleviate or remove this problem altogether, such as synchronization of the pulse and acquisition frequencies to integer proportions in both single-pulse^18^ and continuous-scan mapping modes,^19^ averaging signals based on the least-common multiple of PT and AT,^20^ or more recently, optimization of the total cycle time for sufficient sampling of the SPR profile in the single-pulse mode.^21^ However, all of these remedies have been directed at the quadrupole ICP–MS instruments, where the synchronization can be theoretically exactly achievable, or at least finely tuned by the appropriate choice of dwell times and settling times of the quadrupole mass analyzer. In contrast, in ICP-TOFMS instruments, inherent data acquisition processes preclude perfect synchronization with the laser pulse events, hence some level of aliasing will be an inevitable artifact of this technique. In TOF analyzers, the AT can only be set as a multiple of some base acquisition time, AT_base_, which is a fixed instrumental quantity, equal to the time it takes for a single spectrum to be acquired. Figure 1 portrays a typical time-dependent signal profile (barring the potential Flicker and Poisson noise contributions for simplicity) and the corresponding data reduction output for samples with uniform elemental distribution. The time evolution of the transient SPR profile is modeled after Van Malderen et al.^22^ as a superposition of two exponential (rising and decaying component) functions as described by eq 1 below

1 $$I\left(t\right)=I_{0}\theta \left(t-t_{0}\right)\left(1-e^{-k_{1}\left(t-t_{0}\right)}\right)e^{-k_{2}\left(t-t_{0}\right)}$$

where *I*_0_ is a constant proportional to peak signal intensity, *k*_1_ and *k*_2_ are dispersion factors, *t*_0_ is the characteristic transport time of the aerosol from the ablation cell to the detector and θ(·) is the Heaviside step function. As shown in Figure 1, the ATs of resulting data acquisition events, which report average signal values of a certain number of spectra (purple cross), do not fit evenly into PT, so that each acquisition event that crosses two adjacent pulse intervals, does so at slightly different offsets to the pulse starting times. As a result, the SPR profile of each pulse is sampled slightly differently and the final data, which is obtained by averaging the acquired data points per pulse intervals (red dots), exhibits deviations from an expected constant value. These deviations, which show up as aliasing patterns in elemental maps, are due to instrumental reasons fundamentally irremovable, as seen in the fluctuations of data points per pixel (red dots) in time. Nonetheless, computer simulations and experiments on standard reference materials (SRMs) show that depending on accepted error levels, a judicious choice of AT can successfully minimize aliasing effects even in ICP-TOFMS measurements. Depending on acceptable error levels, herein calculated as the relative standard deviation (RSD) of the signal, substantial improvements in image quality can be achieved while minimizing the redundant accumulation of acquired data points. Still, the sampling of the SPR profile should not be too sparse. While in principle, a single acquisition per PT would be sufficient, this setting produces aliasing patterns visible on longer time scales and is hence not recommended.

**Figure 1:**
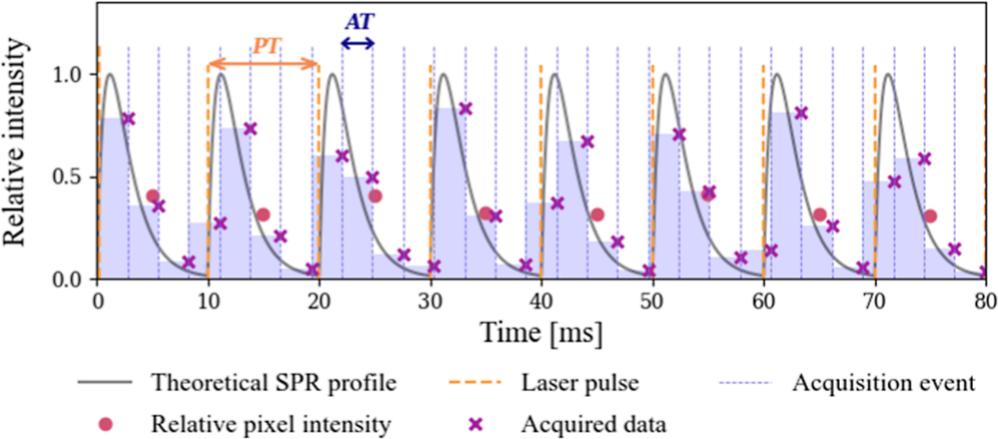
A schematic representation of the LA-ICP-TOFMS data acquisition protocol and the problem of aliasing. PT = pulse time, AT = acquisition time.

## Results and Discussion

### Experimental Measurements of LA-ICP-MS

The LA-ICP-MS experiments were carried out on the NIST SRM 610 glass standard^23^ using an Analyte G2 193 nm ArF* excimer laser ablation system (Teledyne Photon Machines Inc., Bozeman, MT) at the National Institute of Chemistry in Ljubljana (NIC). The LA system equipped with a HelEx II standard two-volume ablation cell was coupled to a Vitesse ICP-TOF-MS (Nu Instruments, Wrexham, UK) via the Aerosol Rapid Introduction System (ARIS) from Teledyne Photon Machines. The complete set of operating parameters for LA-ICP-MS mapping experiments is summarized in Table 1.

**Table 1:** Instrumental Parameters Used at the NIC Laser Ablation System Coupled with TOF-ICP-MS

LA (Analyte G2, ARIS)	
wavelength (nm)	193
laser fluence (J cm^–2^)	3.5
repetition rate (Hz)	100
scanning mode	line scanning
dosage (shots per pixel)	1
washout time (ms)	ca. 10
beam size (μm)	10
mask shape	square
He carrier flow rate (L min^–1^) cup|cell	0.3|0.3

ICP–MS (Vitesse)	
RF power (W)	1300
auxiliary gas flow (L min^–1^)	2
coolant flow (L min^–1^)	13
nebulizer flow (L min^–1^)	1.2
reaction cell gas (mL min^–1^)	15 (He)/6 (H_2_)

### Taming Aliasing in ICP-TOFMS Experiments

Experimental measurements comprising ten adjacent line scans and complementary computer simulations were performed, both yielding elemental maps with dimensions of roughly 10×100 pixels. In all (theoretical and experimental) cases, RR was set to 100 Hz, which amounts to PT = 10 ms. Theoretical signal was obtained by least-squares fitting of eq 1 to SPR profiles from previous experiments in the lab. The interval of the fitted curve corresponding to the full width at 1% of the maximum (FW0.01M) was used to define the SPR profile in a given pulse interval of 10 ms (hence the chosen RR). The AT was varied between the instrumental lower limit of 3AT_base_, with AT_base_ = 26.67 μs, and approximately 8 ms in the upper limit to ensure that all pulse intervals get sampled at least once. This range of values ensures that at least hundreds of SPR profiles fit into a single scan, which allows for the investigation of many possible experimental conditions prone to aliasing. For computer simulations, all integer multiples of AT_base_ in the [3AT_base_, 8 ms] interval were considered, while experimental measurements were carried out at a selected set of times, equal to 16, 52, 76, 104, 156, 208, and 268 times AT_base_, which roughly uniformly covers the whole interval.

Figure 2a shows the calculated RSD values from the pulse-resolved averaging of acquired data, with AT varying between 3AT_base_ ≤ AT < PT. As expected, the RSD initially increases with increasing AT, since the number of acquired data points decrease and their location with respect to the SPR profile is increasingly more random. In certain subintervals, however, the RSD decreases sharply, acquiring local minima. It is instructive to examine the distance of the PT-to-AT ratio to the nearest integer, D_PA_, defined in eq 2 as

2 $$D_{PA}=\min\left\{\frac{PT}{AT}-\lfloor \frac{PT}{AT}\rfloor ,\lceil \frac{PT}{AT}\rceil -\frac{PT}{AT}\right\}$$

where ⌊·⌋ and ⌈·⌉ are the floor and ceiling functions, respectively. The local minima of RSD closely correspond to the minima of D_PA_ (Figure 2b), or in other words, whenever the laser pulses and data acquisition are almost exactly synchronized. This is clearly depicted by vertical dotted lines in Figure 2, located at points where D_PA_ = 0, which correspond to hypothetical ratios PT/AT = 1, 2, 3, 4, 5, etc., around which the RSD steeply decreases. Interestingly, the local maxima in RSD do not occur exactly close to the maximally misaligned time scales (D_PA_ = 0.5), but with a slight offset to lower ATs, which is probably a consequence of the skewness of simulated (and real) SPR profiles. As mentioned before and also shown in the inset in Figure 2a, complete synchronization is not achievable for TOF instruments, since the AT can only be chosen from a discrete set of times. As a result, the RSD will always stay finite, which is unlike in quadrupoles, where in principle, complete elimination of aliasing is a possibility (compare Figures 2a to 3 in van Elteren et al.,^19^ where RSD due to aliasing goes to zero at D_PA_ = 0). One practical approach to deal with this problem is to set a threshold RSD_max_ (for example RSD_max_ = 5%) which we deem acceptable for aliasing noise in our experiments. If we know the SPR profile, we can estimate the location of RSD_max_ from simulations and determine AT_min_ as the lowest multiple of AT_base_ where RSD = RSD_max_. Then, the admissible ATs may be located somewhere inside the interval [AT_min_, PT]. By considering this approach when optimizing the quality of elemental maps, we may go in either direction when choosing the suitable AT. By bringing it down toward AT_min_, the sampling of the SPR profile improves and the RSD decreases, although at the cost of increasing the number of acquired data points per pixel. As shown in Table 2, lowering the AT drastically enlarges the acquired data files-by one to 2 orders of magnitude (in kilobytes) for a simple 10 s line scan. This can quickly result in accumulation of several GBs of additional data with redundant information content when mapping large sample sections containing hundreds to thousands of lines.

**Figure 2:**
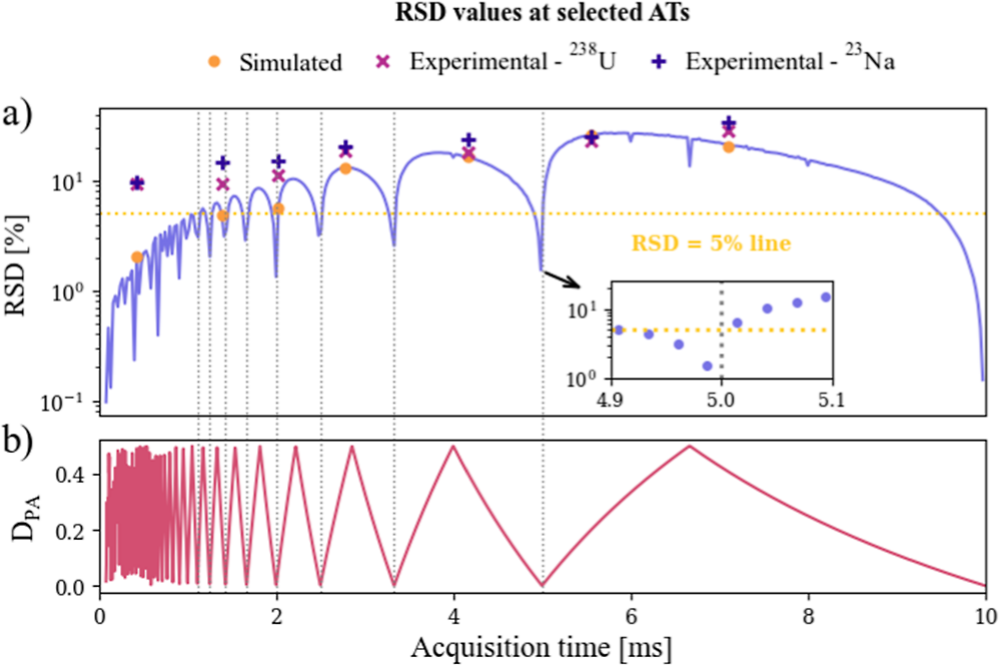
(a) Simulated and experimental RSD values of pulse-averaged signals for LA-ICP-TOFMS measurements at different ATs and PT = 10 ms. The blue curve represents the simulated results for all possible ATs within the 0–10 ms interval, while the orange dots show the simulated data at the same ATs as selected in the experiment. (b) The distance of the PT/AT ratio to the nearest integer. Vertical dotted lines connect the local minima in both plots. See main text for more details.

**Figure 3:**
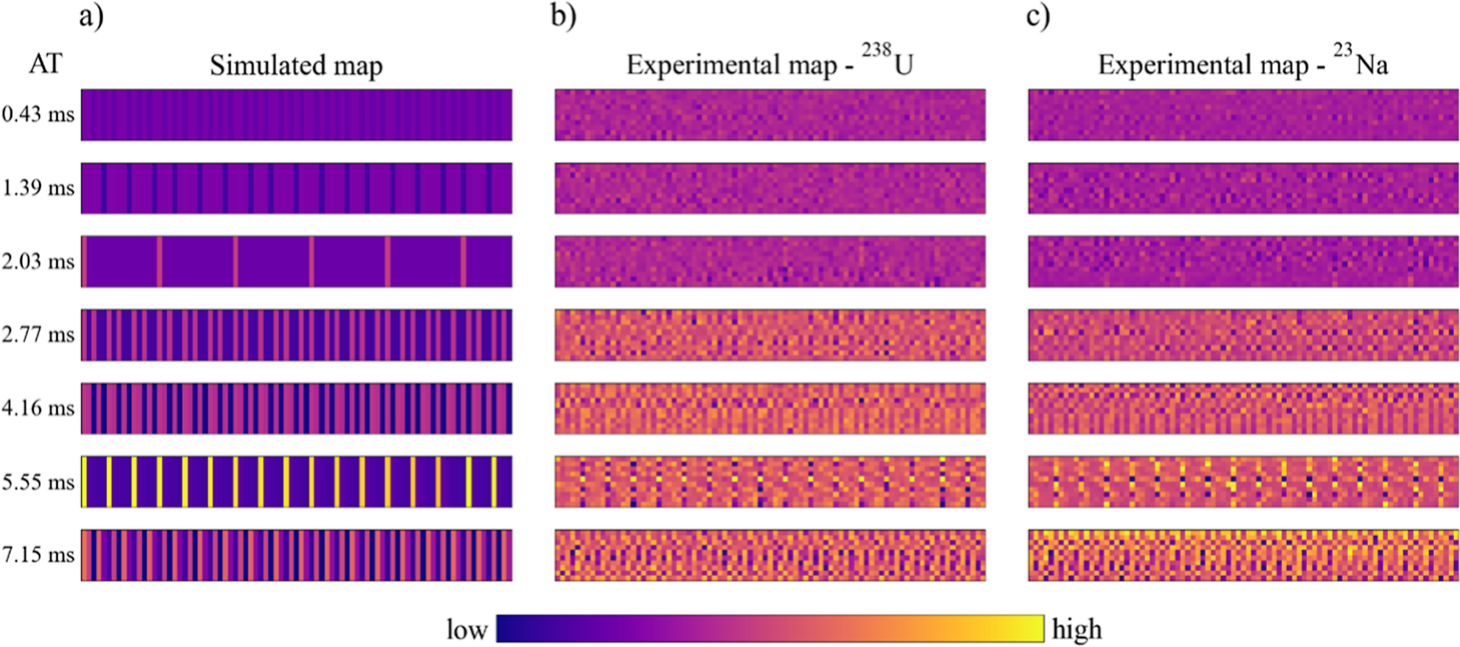
Simulated and experimentally measured elemental maps for different acquisition times. (a) Simulated elemental maps of a hypothetical sample with uniform elemental concentration profile. (b) Experimental maps for ^238^U. (c) Experimental maps for ^23^Na. All maps consist of ten 85-pixel long lines, vertically stacked into images.

**Table 2:** Comparison of the RSD Values and the Sizes of the Acquired Data Files, for Different Acquisition Times and a Total Scan Time of 10 s

AT [ms]	RSD [%]	data size^a^ (10 s line scan)
0.43	2.01	1.1 MB
1.39	4.91	352 kB
2.03	5.72	241 kB
2.77	13.19	176 kB
4.16	16.43	117 kB
5.55	26.21	88 kB
7.15	20.63	68 kB

a The simulated size of a csv file with with acquired data in the form of *time, intensity* columns.

Alternatively, increasing the AT and setting it as close as possible to PT or the integer ratios of PT/AT also lowers the RSD. However, additional care has to be taken in that case, since longer ATs produce very long and/or shallow aliasing patterns while still retaining low RSDs (see Figures S1 and S2 for theoretical simulations with ATs close to the PT). For example, as PT/AT approaches 1, the two time scales are almost completely synchronized, such that PT ≈ *m*(PT–AT) for some m∈N . In terms of AT_base_, for the highest possible AT < PT, the aliasing artifact will repeat according to eq 3 roughly every

3 $$m=\frac{PT}{PT-{AT}_{base}\lfloor \frac{PT}{{AT}_{base}}\rfloor }$$

pixels. Based on the above formula, an estimate can be made that for PT = 10 ms and AT_base_ = 26.67 μs, a low-frequency drift pattern will recur every *m* = 393 pixels at the high end of AT values. Moreover, while not explored thoroughly in this work, the mismatch between the AT and the PT will be fundamentally larger with shorter SPR profiles (corresponding to lower PTs in the single-pulse mode) and we expect more pronounced aliasing in those cases. While simulations predict that the overall RSD variation with AT is very similar, the differences with respect to the pulse length are more conspicuous at low ATs, where signals with lower PTs (correspondingly, higher RRs) are comparatively undersampled, resulting in higher RSDs in that range (for precise trends, the reader is referred to Figures S3 and S4 in the Supporting Information). Finally, the AT > PT scenario is undesirable because it produces pixels with missing values whenever data acquisition skips a whole pulse interval (see Figure S2). In that case, one possible remedy is imputation using any of the known methods (e.g., zero, mean or local mean imputation based on neighboring pixels). However, this strategy is not guaranteed to accurately represent the true, unobserved data.

Experimental LA-ICP-TOFMS measurements on NIST SRMs were observed to be in good agreement with theoretical investigations presented above. In the Figures 2a and 3, comparisons are made between simulated elemental maps and two experimental maps, one for ^23^Na and one for ^238^U, which were chosen for their relatively stable SPR profiles,^24,25^ comparable in shape to our theoretical SPR. The increasing RSD with AT (Figure 2) closely correlates with periodic patterns in the images with increasingly pronounced contrast and very similar progressions in the visual appearance. In the experimental maps (Figure 3b,c) at the lowest three ATs, the Poisson noise still has a dominant effect, which is also the reason why their RSDs are about 5–10% higher than the simulated ones (Figure 3a). In all three cases, the aliasing is visually almost unnoticeable and the images have comparable appearance, except for the third row from top, with AT ≈ 2.03 ms (76AT_base_), where one-pixel vertical lines are faintly visible for the two elements. This observation agrees well with Figure 2, where RSD for the second and third row lies close to a local minimum. For the highest four ATs, aliasing is clearly more visible and steadily increases for both elements. This trend is also predicted by simulations, as can be observed from Figure 2, where the theoretical RSD points lie on the concave regions closer to the local maximum values. Considering the final processed data presented in Figure 3 and the preceding discussion on instrumental and theoretical aspects regarding this work, we may regard the choice of AT = 76AT_base_, which is slightly above PT/5, as a good compromise between minimizing aliasing noise and reducing the amount of acquired data, with 4 acquired data points per PT and RSD close to 5%.

Finally, although the present work has focused primarily on homogeneously distributed elemental maps, it is important to emphasize that most routine LA-ICP-MS applications involve samples with pronounced spatial heterogeneity. A comprehensive investigation of aliasing effects in nonhomogeneous maps would require systematic consideration of features with varying geometries, dimensions, contrasts, and spatial distributions, which is beyond the scope of the present study. Nevertheless, the qualitative influence of aliasing on spatial features is illustrated by the simulation shown in Figure 4. The simulated sample consists of three square-shaped features of progressively decreasing size and linearly decreasing intensity from left to right, embedded within a surrounding region of lower relative intensity (Figure 4a). At low AT (Figure 4b), aliasing artifacts remain comparatively subtle; however, the periodic signal modulation becomes visually amplified when intersecting spatial features or feature boundaries. As a result, the aliasing pattern may introduce apparent intensity fluctuations or distortions that could complicate interpretation of the recorded elemental distribution. Despite this, at AT = 76AT_base_, the simulated features remain largely preserved, suggesting that moderate aliasing at this acquisition time could still be mitigated effectively through appropriate postprocessing or algorithmic correction procedures. At higher AT (Figure 4c), the effects of aliasing become substantially more pronounced. The periodic sampling distortion increasingly alters feature morphology and edge definition, while the weakest feature partially merges with the background signal. These observations demonstrate that inappropriate AT selection can affect not only the faithful representation of concentration profiles, but also the accurate reconstruction of spatial feature geometry and contrast. Consequently, careful optimization of AT is critical for minimizing aliasing-induced distortions in LA-ICP-MS imaging, particularly in applications involving fine spatial structures or low-contrast features.

**Figure 4:**
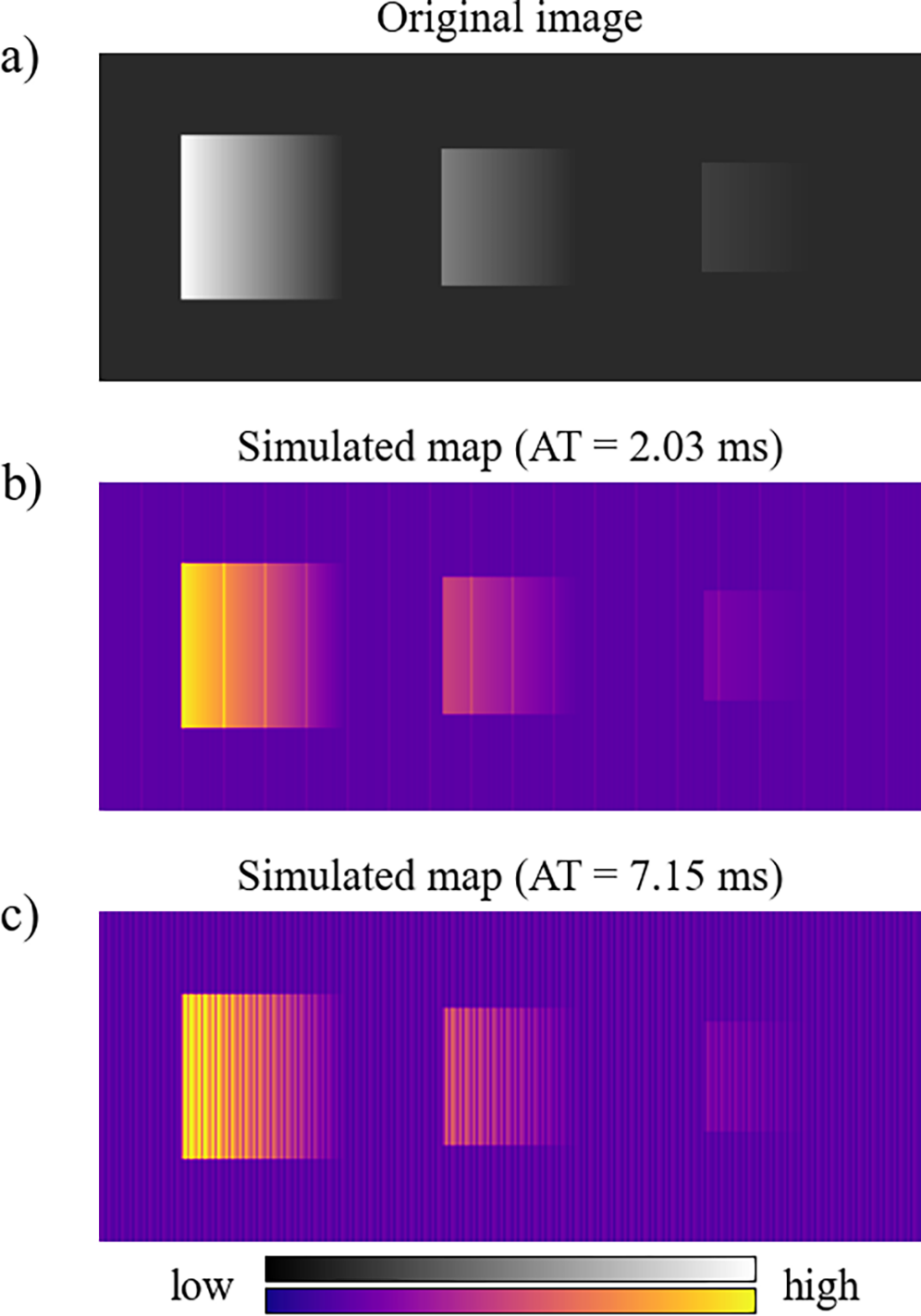
Simulated influence of aliasing on spatial features in LA-ICP-TOFMS maps at PT = 10 ms. (a) Original image, representing a ground-truth elemental distribution in a virtual sample. (b) Simulated map acquired at low acquisition time (AT = 76AT_base_ = 2.03 ms). (c) Simulated map acquired at high AT (238AT_base_ = 7.15 ms). See main text for additional information on the figure.

## Conclusions

We have shown that addressing the problem of aliasing is as critically important in LA-ICP-TOFMS as in quadrupole instruments. Due to the inherent constraints of data acquisition, complete synchronization between the laser pulse and data acquisition events is not achievable for TOF instruments. Numerical simulations provide a useful tool for determining the extent of error induced by temporal mismatch between the two time scales. Unlike in quadrupole instruments, where aliasing can be eliminated, the choice of acquisition time in LA-ICP-TOFMS is a matter of compromise between minimizing the dispersion of the signal and the amount of acquired data. While shorter acquisition times invariably lead to better image quality due to finer and more accurate sampling of the transient signal, the improvements may not necessarily warrant the price of additional data acquisition. By deciding on a threshold for acceptable RSD levels, higher acquisition times with coarser sampling can produce images of comparable quality, as shown in experimental mapping results. In line with previous research on quadrupole mapping, tuning the acquisition time close to an integer quotient of pulse time is also a viable strategy for TOF-based mapping. Based on the simulations and experimental measurements, we suggest that acquiring data 5 times faster than the pulse time or the pixel acquisition frequency should suffice to keep aliasing at a minimum.

## Supplementary Material



## Notes

Includes four additional figures (PDF)
