# Supplementary material for: Aliasing Artifacts in Multielemental LA-ICP-TOFMS Mapping: An Old Problem Revisited
Source: Anal Chem. 2026 Jun 3;98(23):16813–8. doi: 10.1021/acs.analchem.6c01023 (PMC13276849; doi:10.1021/acs.analchem.6c01023)
Supplement: Supplementary file 1 [file ac6c01023_si_001.pdf]

SUPPORTING INFORMATION

Aliasing Artifacts in Multielemental LA-ICP-TOFMS Mapping: An Old Problem Revisited

Filip Cernatič,[1*] Kristina Mervič,[2] Lukas Schlatt,[3] and Martin Šala[1]*
[1]National Institute of Chemistry, Department of Analytical Chemistry, SI-1000 Ljubljana, Slovenia
[2] National Institute of Chemistry, Department of Catalysis and Chemical Reaction Engineering, SI-1000 Ljubljana, Slovenia
[3]Nu Instruments, LL13 9XS Wrexham, United Kingdom

Table of Figures

Figure S1. Simulated elemental maps for the pulse time (and SPR profile) of 10ms and two nearest acquisition times. 3

Figure S2. Simulated elemental maps for the pulse time (and SPR profile) of 1ms and two nearest acquisition times. White vertical lines in the bottom map represent pixels with missing data. 4

Figure S3. Simulated RSD values of pulse-averaged signals for LA-ICP-TOFMS measurements at different ATs and RRs.. 5

Figure S4. Simulated RSD values as in Figure S1, plotted together with respect to admissible acquisition times in the range of [3ATbase, 5ms). 6

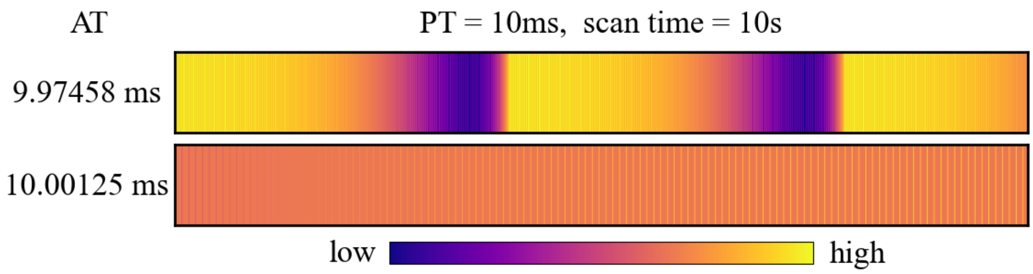

Figure S1. Simulated elemental maps for the pulse time (and SPR profile) of 10 ms and two nearest acquisition times.

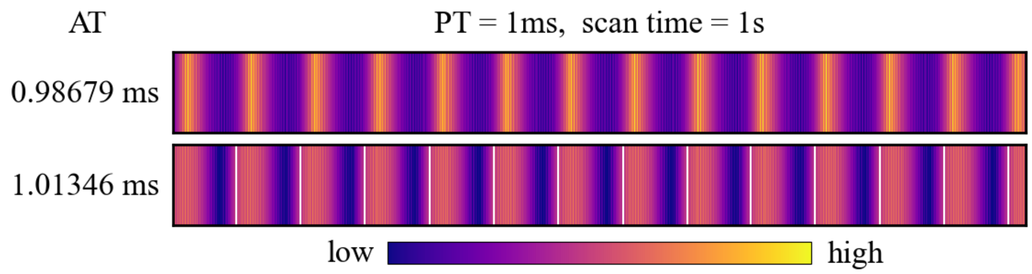

Figure S2. Simulated elemental maps for the pulse time (and SPR profile) of 1 ms and two nearest acquisition times. White vertical lines in the bottom map represent pixels with missing data.

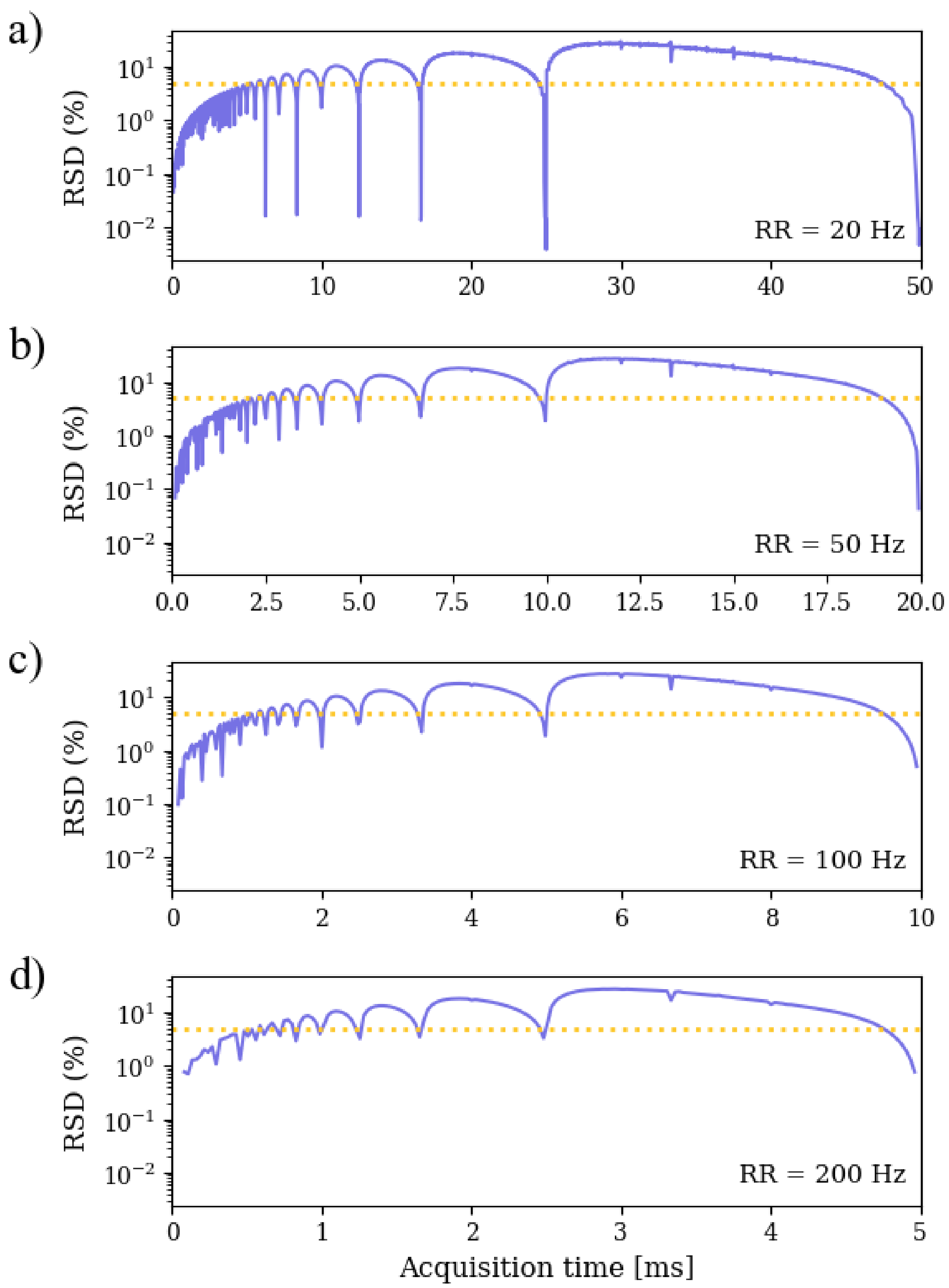

Figure S3. Simulated RSD values of pulse-averaged signals for LA-ICP-TOFMS measurements at different ATs and RRs corresponding to the PTs of (a) 50 ms, (b) 20 ms, (c) 10 ms and (d) 5 ms. The dotted yellow lines correspond to RSD=5% in each subplot.

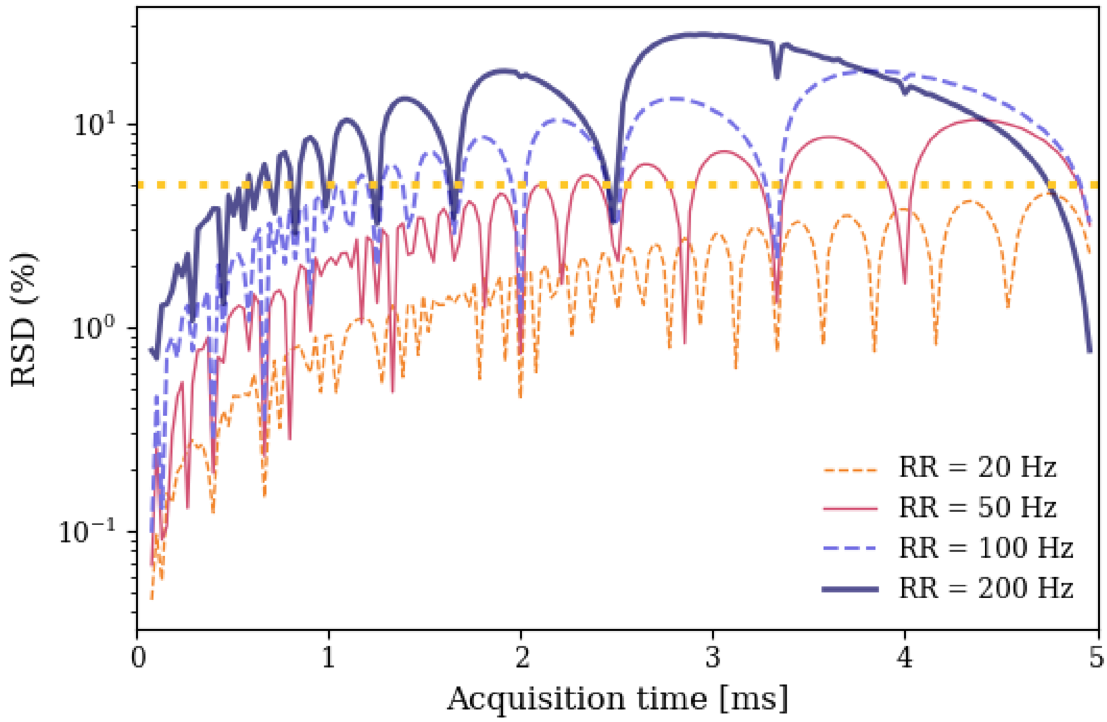

Figure S4. Simulated RSD values as in Figure S1, plotted together with respect to admissible acquisition times in the range of $[3AT_{base}, 5\text{ ms})$.
